# Effect of erythropoietin on level of circulating endothelial progenitor cells and outcome in patients after acute ischemic stroke

**DOI:** 10.1186/cc10002

**Published:** 2011-01-26

**Authors:** Hon-Kan Yip, Tzu-Hsien Tsai, Hung-Sheng Lin, Shu-Fang Chen, Cheuk-Kwan Sun, Steve Leu, Chun-Man Yuen, Teng-Yeow Tan, Min-Yu Lan, Chia-Wei Liou, Cheng-Hsien Lu, Wen-Neng Chang

**Affiliations:** 1 Division of Cardiology, Department of Internal Medicine, Chang Gung Memorial Hospital - Kaohsiung Medical Center, Chang Gung University College of Medicine, 123 Ta-Pei Road, Niaosong District, Kaohsiung City, 833, Taiwan; 2 Center for Translational Research in Biomedical Sciences, Chang Gung Memorial Hospital - Kaohsiung Medical Center, Chang Gung University College of Medicine, 123 Ta-Pei Road, Niaosong District, Kaohsiung City, 833, Taiwan; 3 Department of Neurology, Chang Gung Memorial Hospital - Kaohsiung Medical Center, Chang Gung University College of Medicine, 123 Ta-Pei Road, Niaosong District, Kaohsiung City, 833, Taiwan; 4 Division of General Surgery, Department of Surgery, Chang Gung Memorial Hospital - Kaohsiung Medical Center, Chang Gung University College of Medicine, 123 Ta-Pei Road, Niaosong District, Kaohsiung City, 833, Taiwan; 5 Division of Neurosurgery, Department of Surgery, Chang Gung Memorial Hospital - Kaohsiung Medical Center, Chang Gung University College of Medicine, 123 Ta-Pei Road, Niaosong District, Kaohsiung City, 833, Taiwan

## Abstract

**Introduction:**

Erythropoietin (EPO) enhances the circulating level of endothelial progenitor cells (EPCs), which has been reported to be associated with prognostic outcome in ischemic stroke (IS) patients. The aim of this study was to evaluate the time course of circulating EPC level and the impact of EPO therapy on EPC level and clinical outcome in patients after acute IS.

**Methods:**

In total, 167 patients were prospectively randomized to receive either EPO therapy (group 1) (5,000 IU each time, subcutaneously) at 48 h and 72 h after acute IS, or serve as placebo (group 2). The circulating level of EPCs (double-stained markers: CD31/CD34 (E_1_), CD62E/CD34 (E_2_) and KDR/CD34 (E_3_)) was determined using flow cytometry at 48 h and on days 7 and 21 after IS. EPC level was also evaluated once in 60 healthy volunteers.

**Results:**

Circulating EPC (E_1 _to E_3_) level at 48 h after IS was remarkably higher in patients than in control subjects (*P *< 0.02). At 48 h and on Day 7 after IS, EPC (E_1 _to E_3_) level did not differ between groups 1 and 2 (all *P *> 0.1). However, by Day 21, EPC (E_1 _to E_3_) level was significantly higher in group 1 than in group 2 (all *P *< 0.03). Additionally, 90-day recurrent stroke rate was notably lower in group 1 compared with group 2 (*P *= 0.022). Multivariate analysis demonstrated that EPO therapy (95% confidence interval (CI), 0.153 to 0.730; *P *= 0.006) and EPC (E3) (95% CI, 0.341 to 0.997; *P *= 0.049) levels were significantly and independently predictive of a reduced 90-day major adverse neurological event (MANE) (defined as recurrent stroke, National Institutes of Health Stroke scale ≥8, or death).

**Conclusions:**

EPO therapy significantly improved circulating EPC level and 90-day MANE.

**Trial registration number:**

ISRCTN: ISRCTN96340690

## Introduction

Stroke, a growing epidemic, remains a leading cause of mortality and disability worldwide [1-3]. Surprisingly, while the epidemiology, etiologies, mechanisms, classification, and prognostic outcomes of ischemic stroke (IS) have been widely investigated for several decades, a safe and effective treatment strategy for patients after acute IS has not been fully developed [4-8].

Recently, thrombolysis using tissue plasminogen activator (tPA), a more aggressive management strategy, has been shown to be effective for some acute IS patients early after the onset of symptoms [9,10]. However, tPA use is hampered by many limitations in daily clinical practice [10-13]. In addition to its narrow indication for only a small number of patients, tPA therapy has been reported to have a relatively high incidence of intracranial bleeding complications [13,14]. The majority of acute IS patients, therefore, are still left without any specific treatment. Hence, finding a safe and effective therapeutic regimen for patients following acute IS, especially those unsuitable for thrombolytic therapy, is of utmost importance for physicians.

Erythropoietin (EPO) was originally used for treating anemic patients of various etiologies, especially for patients with uremia. Interestingly, in addition to its role in normalizing erythropoiesis, EPO has been clearly shown to exert a myocardial protective effect against ischemia-related damage [15-17]. In contrast, the neuroprotective effect of EPO after acute IS is not well-documented and the results are inconsistent [18-20]. The mechanisms underlying the anti-ischemic action of EPO have been proposed to involve anti-apoptotic processes [15,16], neovascularization, mobilization of endothelial progenitor cells (EPCs), and angiogenesis [21-23]. An increase in circulating levels of EPCs in patients after acute IS has been demonstrated to be strongly associated with favorable clinical outcomes in our recent study [24]. Accordingly, we proposed that other than its role in protecting myocardium against ischemic insult, EPO therapy may enhance the circulating EPC level and improve neurological function and clinical outcome in patients after acute IS.

## Materials and methods

### Study design

This clinical trial was approved by the Institutional Review Committee on Human Research in Chang Gung Memorial Hospital (No 96-1381A) in 2007 and conducted at Kaohsiung Chang Gung Memorial Hospital.

This was a prospective, randomized, and placebo-controlled trial. The primary objective was to evaluate the safety and efficacy of two consecutive doses of EPO (Epoetin beta, Roche, Basel, Switzerland) (5,000 IU each time, subcutaneously) administered at 48 h and 72 h after acute IS in improving the 90-day combined endpoint of recurrent stroke or death. The secondary objective of this study was to establish the time course of circulating levels of EPCs in patients after acute IS and the ability of two doses of EPO in enhancing circulating EPC level. In addition, this study's intent was to assess the impact of EPO therapy on improving the combined adverse neurological event (MANE) (defined as recurrent stroke, National Institutes of Health Stroke Scale (NIHSS) ≥8, or death). The definition of the MANE was based on our recent reports [8,24]. Instead of EPO, the placebo-control subjects received a 1 mL normal saline subcutaneous injection at 48 h and 72 h after acute IS. Additionally, a neurologist blinded to the treatment allocation assessed the outcomes. The medication (trial agent) was given by a clinician blinded to the patients' clinical condition. Patients who had a history of allergy to EPO, hematological disorders including myeloproliferative disorder, leukemia, thrombocythemia, polycythemia, past history of deep vein thrombosis, abnormal elevation of hemoglobin (male >14.5 gm/dL; female >13.5 gm/dL) were excluded from this trial.

### Calculation of sample size for specific objective

The study included consecutively admitted acute IS patients at a single facility between October 2008 and March 2010. For the primary objective of the study, an estimated sample size of 106 study patients in each group was based on the effective size with an α = 0.05, a power of 80%, an anticipation of a combined end point of 14.0% in placebo control vs. 4.0% with EPO therapy. For the secondary objective of this study, an estimated sample size of 93 study patients in each group was based on the effective size with an α = 0.05, a power of 80%, an average difference in circulating level of EPCs between the EPO therapy and placebo-control group of 0.32%, and a standard deviation of circulating level of EPCs in EPO therapy was 0.7%. A 20% rate of protocol violations and incomplete follow-up was assumed. The calculation of sample size for specific objective was based on our recent report [24].

### Definition and exclusion criteria

Stroke was defined as sudden onset of loss of global or focal cerebral function persisting for more than 24 h. Patients of any age with acute IS were eligible. Inclusion criteria included a scoring of >2 on the NIHSS (scores up to 8 indicate moderate neurological status disability) and a time window of ≤48 h from onset of symptoms to blood sampling (at 48 h after IS) and study drug administration (time to treatment just after blood sampling). Patients with a history of the following were excluded from the study: intracranial hemorrhage, surgery or trauma within the preceding three months, abnormal liver function, hematology disorders, renal insufficiency (serum creatinine >1.5 mg/dL), malignancy, febrile disorders, acute or chronic inflammatory disease at study entry, liver cirrhosis, atrial fibrillation, congestive heart failure, contraindications for Magnetic Resonance Imaging (MRI) examination, no evidence of acute IS by MRI study, myeloproliferative disorder, antibodies or allergies to EPO, pregnancy, tPA therapy for acute IS, or a hemoglobin level >15.0 gm/dL.

**Figure 1:**
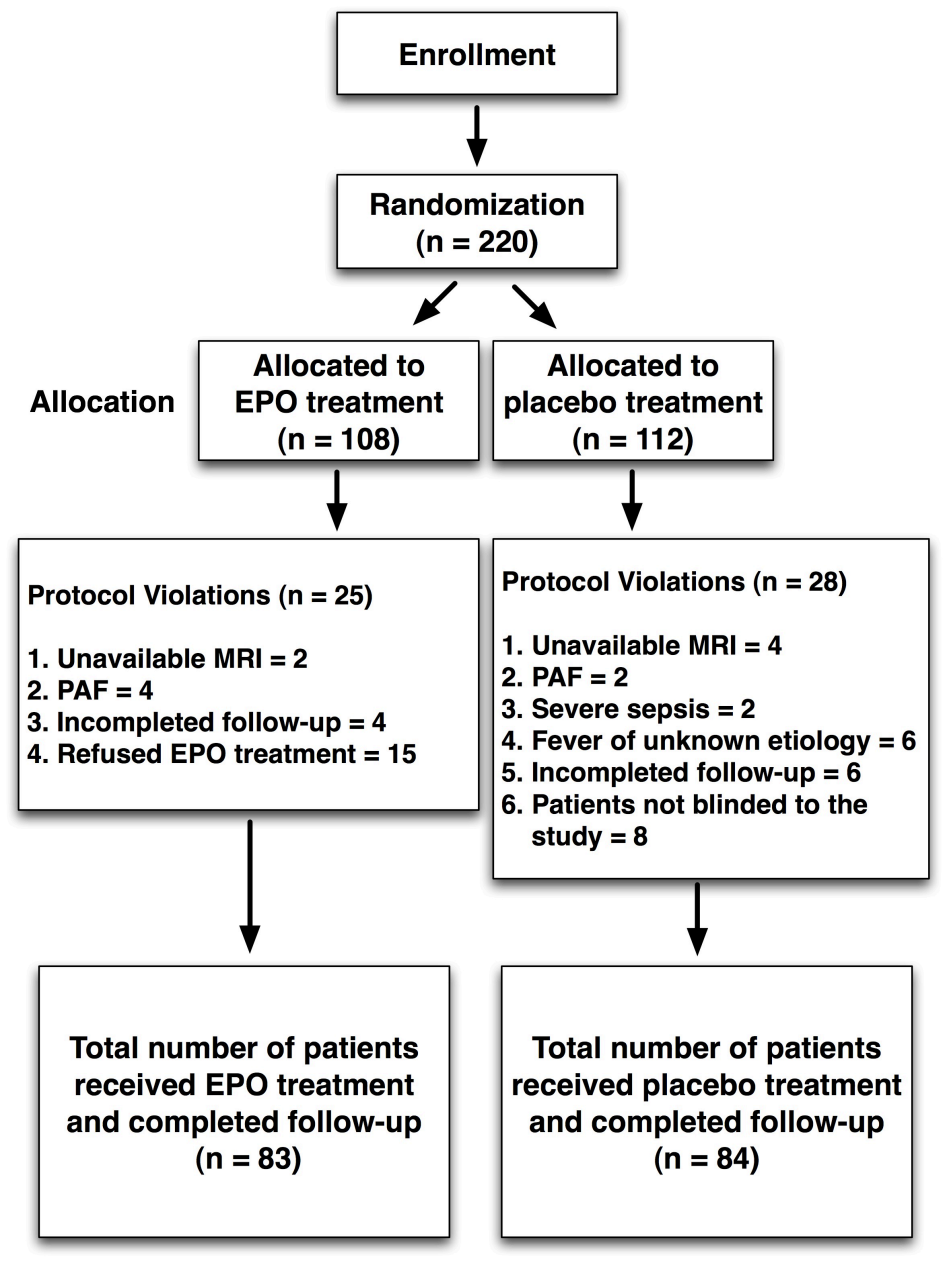
**Schematic overview of the trial protocol**. EPO, erythropoietin; MRI, magnetic resonance imaging; PAF, paroxysmal atrial fibrillation.

An overview of the study protocol of this clinical trial is shown in Figure 1. From October 2008 through March 2010, consecutive patients with acute IS were enrolled by the responsible neurologists at the institute. Patients were randomly assigned to different treatment groups after giving informed consent. Over a period of 18 months, 230 consecutive patients with IS occurring less than 48 h prior to blood sampling were recruited.

Twenty-five (23.1%) of the 108 EPO-treated (group 1) patients were excluded due to unavailable MRI data (two patients), paroxysmal atrial fibrillation (four patients), refused EPO therapy (fifteen patients), or incomplete follow-up (four patients) that occurred later after the IS. Therefore, the remaining 83 patients constituted the EPO therapy group (group 1). Twenty (17.9%) of the 112 patients in the placebo control group (group 2) were excluded due to unavailable MRI data (four patients), paroxysmal atrial fibrillation (two patients), fever and sepsis (two patients), fever of unknown etiology (six patients), or incomplete follow-up (six patients) that occurred later after the IS. Additionally, eight patients (7.1%) who insisted on knowing the type of therapeutic drug after enrollment were also excluded even though their blood samples were collected. Therefore, the remaining 84 patients constituted group 2 (placebo control) of this study.

Sixty age- and gender-matched healthy volunteers were also studied for circulating level of EPCs. Informed consent was obtained from all study subjects.

### Neurological assessment

Evaluation of the physical function and degree of neurological impairment in the stroke patients was based on the National Institutes of Health Stroke Scale (NIHSS) [25] during the acute (at 48 h), convalescent (on Day 21), and chronic (Day 90) phases of stroke by neurologists blinded to the treatment allocation (double-blind study). Moderate neurological impairment (that is, neurological sequelae that requires partial support in daily activities) was defined as a score of ≥8 on NIHSS, a modified criteria reported previously [4]. In addition to NIHSS, assessments only during admission included functional measures, Barthel Index [26] [range from 100 (no deficit) to 0 (complete dependence or death)], and modified Rankin Scale score [27] (range from 0 (no residual symptoms) to 6 (indicating death)).

### Imaging studies and laboratory investigations

In addition to full clinical assessment, other examinations performed also included chest X-ray film, routine brain computed tomography, duplex scanning of the carotid arteries, and routine cardiac analysis by 12-lead electrocardiogram and echocardiography. Moreover, white blood cell (WBC) count, red blood cell (RBC) count, hemoglobin, and biochemical data were acquired at 48 h and on days 7 and 21 after acute IS.

The radiological diagnosis of acute IS included brain computed tomography showing a new finding of low attenuation density in focal or diffuse brain area; or MRI examination showing area(s) of high intensity (bright spots) on diffusion weighted image (DWI) MRI or lower intensity on apparent diffusion coefficient (ADC) value MRI.

### Blood sampling and assessment of circulating EPC level by flow cytometry

Blood samples were obtained at 48 h (acute phase) and on days 7 (recovery phase) and 21 (convalescent phase) after IS at 9.00 a.m. for assessment of the serial changes in circulating level of EPCs in IS patients. Blood samples were also obtained in control subjects who participated in a health screening program in our Health Clinic once at 9.00 a.m.

Ten milliliters of blood was drawn from the antecubital vein into a vacutainer containing 3.8% buffered sodium heparin. Mononuclear cells (MNCs) were then isolated by density-gradient centrifugation of Ficoll 400 (Ficoll-Plaque™ plus, Amersham Biosciences, Uppsala, Sweden), based on our recent report [24]. The MNCs were washed twice with phosphate buffered saline (PBS) and centrifuged before incubation with 1 mL blocking buffer for 30 minutes at 4°C. Cell variability of >95.0% was noted in each group.

A flow cytometric method for identification of EPCs derived from peripheral blood has been reported in our recent studies and also those by others [24,28,29]. Briefly, the isolated MNCs (4 × 10^5^) were incubated for 30 minutes at 4°C in a dark room with monoclonal antibodies against kinase insert domain-conjugating receptor (KDR) (Sigma, St. Louis, MO, USA), the fluorescein isothiocyanate (FITC)-conjugated CD34 and the phycoerythrin (PE)-conjugated CD31, and CD62E (Becton Dickinson, San Jose, CA, USA) to determine the EPC surface markers of CD31/CD34 (E_1_), CD62E/CD34 (E_2_), and KDR/CD34 (E_3_), The control ligand (IgG-PE conjugate) was used to detect any nonspecific association and define a threshold for glycoprotein binding. For analysis of KDR, the MNCs were further incubated with PE-conjugated anti-mouse antibody made in goat. After staining, the MNCs were fixed in 1% of paraformaldehyde. Quantitative two-colored flow cytometric analysis was performed using a fluorescence-activated cell sorter (FACSCalibur™ system; Beckmen Coulter, Brea, CA, USA). Each analysis included 30,000 cells per sample. The assays for EPCs (E_1 to 3_) in each sample were performed in duplicate, with the mean level reported.

Intra-assay variability based on repeated measurement of the same blood sample was low with a mean coefficient of variance being 3.9% and 3.6% in stroke patients and in normal subjects, respectively.

### Medications

Aspirin was the first choice for acute stroke patients unless they were allergic or intolerant to aspirin, including a history of peptic ulcer or upper gastro-intestinal tract bleeding during aspirin therapy. Clopidogrel was used in patients intolerant to aspirin therapy. Other commonly used drugs included statins, angiotensin converting enzyme inhibitors (ACEIs)/angiotensin II type I receptor blockers (ARB), diuretics, calcium channel blocking agents, and beta blockers.

### Statistical analysis

Chi-square test or Fischer's exact test was used where appropriate. Comparisons of means were performed using Student *t-*test. Continuous variables at three time points in the three groups were compared using repeated measure of ANOVA followed by Tukey multiple comparison procedure. Multivariate logistic regression analysis was utilized for identifying the independent predictors of EPCs level and prognostic outcomes. Statistical analysis was performed using SAS statistical software for Windows version 8.2 (SAS institute, Cary, NC, USA). A value of *P *< 0.05 was considered statistically significant.

## Results

### Baseline characteristics and laboratory findings of study patients and healthy controls

Table 1 displays the baseline demographic and laboratory findings of both IS patients (that is, group 1 = EPO-treated group, group 2 = placebo control) and healthy controls. There were no significant differences in terms of age, gender, body mass index, diastolic blood pressure (DBP), total cholesterol level, low-density lipoprotein (LDL), serum creatinine level, RBC count, hemoglobin, or hematocrit level between three groups. However, high-density lipoprotein (HDL) was notably lower in IS patients than in healthy controls. In contrast, WBC count and systolic blood pressure (SBP) were remarkably higher in groups 1 and 2 of IS patients compared with the control subjects. Moreover, the level of circulating EPCs (E_1 to 3_) was substantially higher in both groups of IS patients than in healthy controls.

**Table 1:** Comparison of baseline characteristics and laboratory findings among three groups

Variables	Group 1 (*n *= 83)†	Group 2 (*n *= 84)†	Healthy Control (*n *= 60)	*P*-value*
Age (y) (mean ± SD),	63.7 ± 11.4	67.0 ± 11.1	64.1 ± 6.0	0.078
Male, % (n)	65.1% (54)	66.7% (56)	65.0% (39)	0.969
Hypertension, % (n)	63.9% (53)	73.8% (62)	--	0.165
Diabetes mellitus, % (n)	37.4% (31)	32.1% (27)	--	0.480
Current smoking, % (n)	36.1% (30)	273.4% (23)	--	0.224
Previous stroke by history, % (n)	24.1% (20)	21.4% (18)	--	0.681
Previous stroke by MRI, % (n)	62.7% (52)	57.1% (48)	--	0.468
Old myocardial infarction, % (n)	8.4% (7)	6.0% (5)	--	0.549
RBC count (×10^6^/μL)	4.74 ± 0.67	4.68 ± 0.68	4.81 ± 0.64	0.561
Hemoglobin (g/dL)	14.0 ± 2.0	14.1 ± 1.8	14.05 ± 1.56	0.963
Hematocrit (%)	41.3 ± 5.9	41.4 ± 6.0	40.9 ± 6.1	0.877
WBC count (×10^3^/μL)	7.82 ± 2.38^a^	7.83 ± 2.37^a^	5.91 ± 1.84^b^	<0.0001
Circulating level of EPCs at 48 h				
CD31/CD34 (%)	1.65 ± 0.91^a^	1.75 ± 1.03^a^	1.13 ± 0.74^b^	0.0003
CD62E/CD34 (%)	1.21 ± 0.86^a^	1.16 ± 0.76^a^	0.93 ± 0.83^b^	0.025
KDR/CD34 (%)	1.34 ± 0.77^a^	1.37 ± 0.89^a^	1.03 ± 0.79^b^	0.03
Total cholesterol level (mg/dL)	186.6 ± 41.2	190.1 ± 42.7	193.2 ± 36.4	0.621
HDL (mg/dL)	44.6 ± 10.8^a^	49.2 ± 17.3^a^	53.8 ± 14.8^b^	0.001
LDL (mg/dL)	116.2 ± 35.7	115.2 ± 39.4	117.2 ± 30.9	0.949
Creatinine (mg/dL)	1.00 ± 0.38	1.02 ± 0.43	1.01 ± 0.24	0.915
BMI (kg/m^2^)	25.1 ± 3.5	24.2 ± 3.9	24.7 ± 3.1	0.225
HbA_1C _level, %	6.73 ± 1.85	6.96 ± 1.88	--	0.468
SBP (mm Hg)	144 ± 20^a^	143 ± 21^a^	136 ± 18^b^	0.031
DBP (mm Hg)	83 ± 12	84 ± 11	85 ± 11	0.231
Significant ECCA stenosis, % (n)	24.1% (20)	17.9%% (15)	--	0.322
Statin therapy	43.4% (36)	45.2% (38)	--	0.808
ACEI/ARB therapy	39.8% (33)	38.1% (32)	--	0.826
EPO therapy-related adverse events				
Allergy	0% (0)	0% (0)		--
Polycythemia	0% (0)	0% (0)		--
Thrombosis event	0% (0)	0% (0)		--

*: by Chi-square test or Fisher's exact test for categorical data; by t-test or one-way ANOVA for continuous data.

Letters (^a, b^) indicate significant difference (at 0.05 level) by Tukey multiple comparison procedure.

†: group 1, with EPO treatment; group 2, without EPO treatment.

Data are expressed as mean ± SD or % (No.) of patients.

ACEI/ARB, angiotensin converting enzyme inhibitor/angiotensin II type I receptor blocker; BMI, body mass index; DBP, diastolic blood pressure; ECCA, extra-cranial carotid artery; EPC, endothelial progenitor cell; EPO, erythropoietin; HbA_1C, _hemoglobin A_1C_; HDL, high-density lipoprotein; LDL, low-density lipoprotein; MRI, magnetic resonance imaging; SBP, systolic blood pressure; RBC, red blood cell; WBC, white blood cell.

The risk factors of cerebrovascular disease, incidence of previous stroke documented by history or MRI, old myocardial infarction, or hemoglobin A_1C _(HbA_1C_) did not differ between group 1 and group 2 patients. Additionally, the incidence of significant extra-cranial carotid artery (ECCA) stenosis (defined as ≥50% stenosis by carotid Doppler measurement) and the status of both statin and ACEI/ARB treatment did not significantly differ between the two groups. Importantly, no side effect of EPO therapy was recorded. This finding indicates that EPO therapy with the regimen of two consecutive dosages of 5,000 IU per patients is likely to be safe.

### Laboratory findings, circulating level of EPCs at three time points, neurological status, and clinical outcome after acute IS

Table 2 shows that there was also no significant difference between the circulating levels of EPCs (E_1 _to E_3_) at 48 h and on Day 7 between both groups of patients. Moreover, the RBC and WBC counts, hemoglobin, and hematocrit on Day 21 were similar among these patients. However, by Day 21, the circulating level of EPCs (E_1 _to E_3_) was substantially higher in group 1 than in group 2.

**Table 2:** Laboratory findings and circulating level of EPCs between IS patients with and without EPO treatment

Variables	Group 1† (*n *= 83)	Group 2† (*n *= 84)	*P-*value*
Circulating level of EPCs at 48 h			
CD31/CD34 (%)	1.65 ± 0.91	1.75 ± 1.03	0.530
CD62E/CD34 (%)	1.21 ± 0.86	1.16 ± 0.76	0.704
KDR/CD34 (%)	1.34 ± 0.76	1.37 ± 0.89	0.791
Circulating level of EPCs on day 7			
CD31/CD34 (%)	1.52 ± 1.06	1.48 ± 0.89	0.801
CD62E/CD34 (%)	1.11 ± 0.76	1.14 ± 0.75	0.855
KDR/CD34 (%)	1.16 ± 0.70	1.24 ± 0.80	0.523
Circulating level of EPCs on day 21			
CD31/CD34 (%)	2.28 ± 1.48	1.64 ± 0.79	0.002
CD62E/CD34 (%)	1.50 ± 1.13	1.14 ± 0.72	0.030
KDR/CD34 (%)	1.81 ± 1.25	1.22 ± 0.71	0.001
RBC count (×10^6^/mL) on day 21	4.56 ± 0.73	4.62 ± 1.16	0.719
Hemoglobin (g/dL) on day 21	13.7 ± 1.9	13.8 ± 3.3	0.746
Hematocrit (%) on day 21	41.0 ± 5.4	39.9 ± 6.4	0.309
WBC count (×10^3^/mL) on day 21	7.68 ± 6.30	7.27 ± 2.47	0.645

*: by t-test.

†: group 1 = with EPO treatment; group 2 = without EPO treatment.

EPC = endothelial progenitor cell; EPO = erythropoietin; RBC = red blood cell; WBC = white blood cell.

The scores of NIHSS, Barthel Index, and modified Ranking Scale score upon presentation (at 48 h after acute IS) did not differ between group 1 and group 2 (Table 3). Additionally, the mean NIHSS score on Day 90 did not differ between group 1 and group 2. However, the incidence of a NIHSS score ≥8 on Day 90 was notably lower in group 1 than in group 2. Furthermore, although the 90-day mortality was similar between the two groups, the incidence of recurrent stroke during a 90-day follow-up was notably lower in group 1 than in group 2. Besides, the incidence of 90-day major adverse neurological event (that is, MANE) was significantly reduced in group 1 compared with group 2.

**Table 3:** Comparisons of neurological status and clinical outcome between IS patients with and without EPO treatment

Variables	Group 1† (*n *= 83)	Group 2† (*n *= 84)	*P-*value*
NIHSS at 48 h	6.78 ± 4.60	7.35 ± 7.55	0.562
Modified Rankin Scale score at 48 h	3.23 ± 1.38	2.89 ± 1.62	0.152
Barthel Index at 48 h	55.7 ± 30.5	59.5 ± 36.0	0.467
NIHSS on day 90	4.27 ± 5.39	5.49 ± 7.77	0.239
Recurrent stroke, % (n)	0% (0)	9.5% (8)	0.007
90-day mortality, % (n)	2.4% (2)	1.2% (1)	0.621
Primary end point, % (n)‡	2.4% (2)	10.7% (9)	0.057
NIHSS ≥ 8.0	14.5% (12)	29.8% (25)	0.017
Combined MANE, % (n)¶	16.9% (14)	36.9% (31)	0.004

*: by Chi-square test or Fisher's exact test for categorical data; by *t*-test for continuous data.

†: group 1 = with EPO treatment; group 2 = without EPO treatment.

‡: defined as 90-day combined recurrent stroke or death.

¶: MANE = major adverse neurological event (defined as NIHSS ≥8, recurrent stroke or death on Day 90 after acute IS).

IS, ischemic stroke; NIHSS, national institutes of health stroke scale.

### Correlation between three individualized neurological assessment scales upon presentation and the circulating level of EPCs

**Figure 2:**
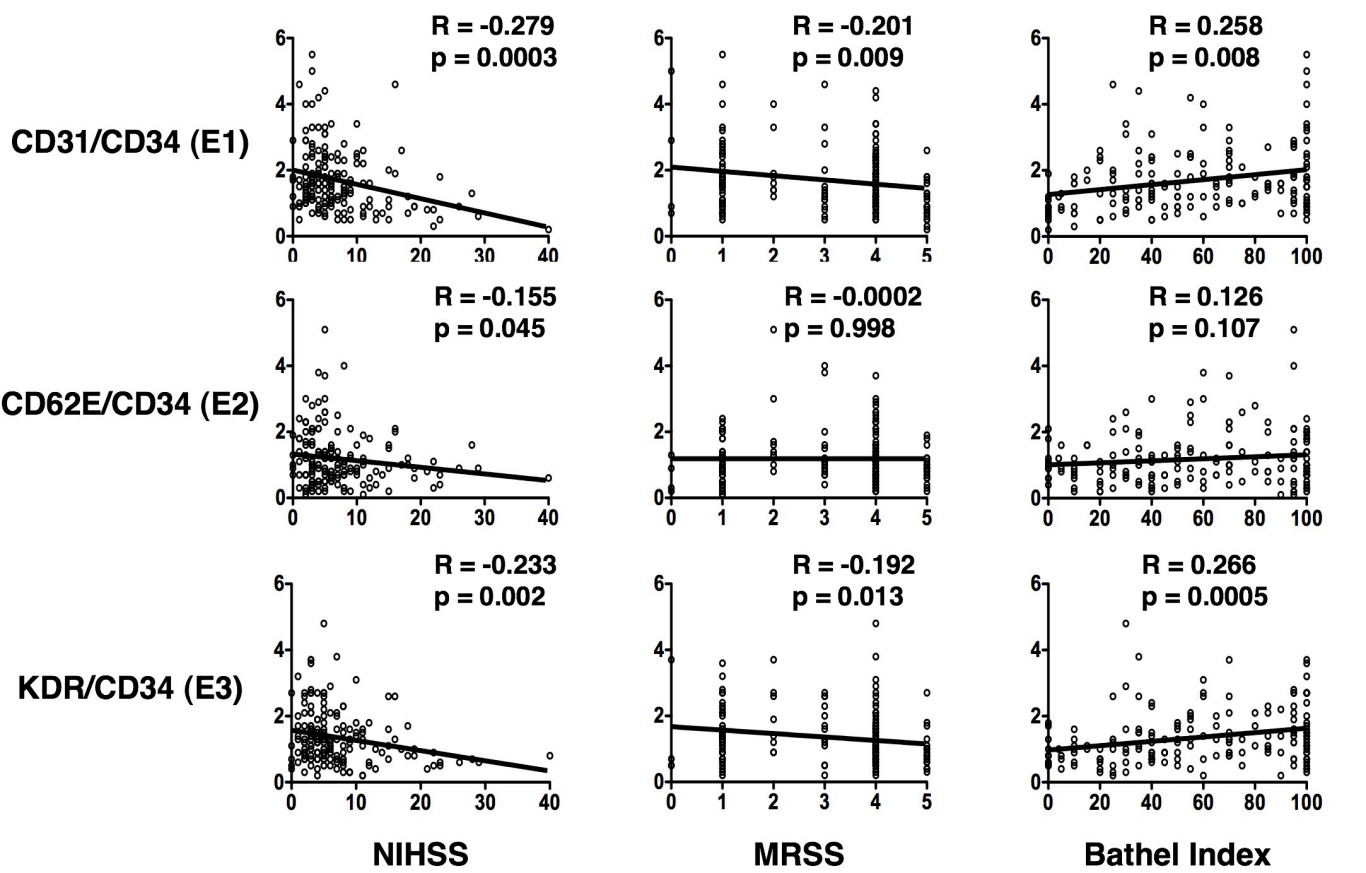
**Correlation between circulating level of endothelial progenitor cells (EPCs) and three individualized neurological assessment scales**. NIHSS, National Institutes of Health Stroke scale; MRSS, modified Ranking Scale score.

The Spearman rank correlation analysis did not identify significant correlation of circulating level of EPCs (E_2_) to either modified Ranking Scale score or to Barthel Index at 48 h after acute IS (Figure 2). On the other hand, a significant negative correlation was noted between the circulating level of EPCs (E_1 _and E_3_) and both modified Ranking Scale and Barthel Index at 48 h after acute IS. Besides, a significant negative correlation also was noted between the circulating level of EPCs (E_1 to _E_3_) and NIHSS at 48 h after acute IS.

### Time course of circulating level of EPCs

The circulating level of EPCs (E_1 _to E_3_) did not significantly alter at the chosen time points (48 h, on days 7 and 21) after acute IS in group 2 patients (Table 4). Consistently, the circulating level of EPCs did not significantly change between at 48 h and on Day 7 after acute IS in group 1 patients. However, the circulating level of EPC (E_1 _to E_3_) was substantially increased on Day 21 compared with that at 48 h and on Day 7 after acute IS in group 1. These findings indicate that the effect of EPO therapy on boosting circulating EPC level was gradually increased after one week and up to a significantly higher level on Day 21 after acute IS.

**Table 4:** Time course of circulating level of EPCs

Variables	At 48 h	On Day 7	On Day 21	*P-*value*
Circulating EPCs in group 1				
CD31/CD34 (%)	1.65 ± 0.91^a^	1.52 ± 1.06^a^	2.28 ± 1.48^b^	<0.0001
CD62E/CD34 (%)	1.21 ± 0.86^a^	1.11 ± 0.76^a^	1.50 ± 1.13^b^	0.0409
KDR/CD34 (%)	1.34 ± 0.76^a^	1.16 ± 0.70^a^	1.81 ± 1.25^b^	0.0001
Circulating EPCs in group 2				
CD31/CD34 (%)	1.75 ± 1.03	1.48 ± 0.89	1.64 ± 0.79	0.071
CD62E/CD34 (%)	1.16 ± 0.76	1.14 ± 0.75	1.14 ± 0.72	0.973
KDR/CD34 (%)	1.37 ± 0.89	1.24 ± 0.80	1.22 ± 0.72	0.267

*: by repeated measure of ANOVA.

Letters (^a, b^) indicate significant difference (at 0.05 level) by Tukey multiple comparison procedure.

### Univariate and multivariate analyses of predictors for 90-day MANE

Univariate analysis of enrollment variables in Tables 1 and 3 demonstrated that serum levels of total cholesterol and LDL were significantly associated with 90-day MANE (Table 5). On the other hand, EPO therapy, increase in circulating level of EPC (E_3_) at 48 h after IS, SBP and DBP were favorable factors strongly predictive of freedom from 90-day MANE. Interestingly, further analysis revealed that the incidence of MANE varied with SBP upon presentation (subgroup (A) ≤135 mmHg, subgroup (B) >135 mmHg to ≤150 mmHg and subgroup (C) >150 mmHg) were 38.3% (*n *= 23/60), 17.0% (9/53), and 24.1% (13/54), respectively. As compared with the other two subgroups, subgroup (B) was significantly associated with a reduction in 90-day MANE (all *P *< 0.05).

**Table 5:** Logistic regression analysis of predictors for combined MANE on Day 90 after ischemic stroke

Variables	Odds Ratio	95% CI	*P-*value
**Univariate**			
Systolic blood pressure*	0.978	0.960 to 0.996	0.017
Diastolic blood pressure*	0.962	0.931 to 0.994	0.019
Total cholesterol level	1.012	1.003 to 1.021	0.006
Low-density lipoprotein	1.013	1.003 to 1.022	0.010
EPO therapy	0.347	0.168 to 0.717	0.004
KDR/CD34	0.609	0.373 to 0.995	0.048
**Multiple Stepwise**			
Total cholesterol level	1.012	1.003 to 1.021	0.010
Systolic blood pressure*	0.979	0.960 to 0.998	0.031
KDR/CD34	0.583	0.341 to 0.997	0.049
EPO therapy	0.334	0.153 to 0.730	0.006

CI, confidence interval; EPO, erythropoietin.

MANE, major adverse neurological event (defined as NIHSS ≥8, recurrent stroke or death on day 90 after acute IS); NIHSS, national institutes of health stroke scale.

* Indicates the continuity of systolic and diastolic blood pressure to be used for statistical analysis but not the history of hypertension.

Multiple stepwise logistic regression analysis demonstrated that total cholesterol level was significantly and independently predictive of 90-day MANE (Table 5). In contrast, SBP, EPO treatment, and circulating EPC (E_3_) level were significantly and independently predictive of improvement in 90-day MANE.

### Univariate and multivariate analyses of predictors for 90-day combined end point (recurrent stroke or death)

Univariate analysis of enrollment variables in Tables 1 and 3 demonstrated that serum levels of creatinine were significantly correlated with 90-day combined end point (Table 6). On the other hand, EPO therapy and serum level of HDL were significantly predictive of freedom from a 90-day combined end point. Multiple stepwise logistic regression analysis demonstrated that EPO treatment and serum level of HDL were significant and independent predictors of freedom from 90-day combined end point (Table 6).

**Table 6:** Predictors for combined death and recurrent stroke on Day 90 after ischemic stroke

Variables	Odds Ratio	95% CI	*P*-value
**Univariate**			
High-density lipoprotein	0.921	0.857 to 0.990	0.026
EPO therapy	0.206	0.043 to 0.983	0.004
Creatinine	3.250	1.045 to 10.109	0.042
**Multiple Stepwise**			
High-density lipoprotein	0.909	0.842 to 0.983	0.016
EPO therapy	0.155	0.031 to 0.772	0.023

CI, confidence interval; EPO, erythropoietin; MANE, major adverse neurological event.

## Discussion

This study, which investigated the safety and efficacy of EPO therapy in boosting the circulating level of EPCs and improving 90-day clinical outcome in patients after acute IS, provides some notable information. First, as compared with the healthy controls, circulating levels of EPCs were remarkably increased in patients after acute IS. Our findings are comparable with those of our recent report [24]. Second, there were no serial changes in circulating EPC level after acute IS. Third, only EPO therapy in the acute phase of IS was associated with an increase in circulating levels of EPC at the convalescent phase of IS. Besides, EPO therapy was significantly associated with a reduction in the incidence of recurrent stroke. Fourth in importance was the fact that, in addition to being without side effects, EPO therapy and increased circulating EPC (E_3, _KDR/CD34) were significantly and independently predictive of a decrease in 90-day MANE.

### Value and level of circulating EPCs after acute IS

Abundant data have shown that EPCs in bone marrow microenvironment [30] migrate to circulation in response to ischemia-related organ dysfunction for angiogenesis [24,31,32]. The present study that demonstrated a marked increase in circulating EPC levels in patients after acute IS compared with the normal controls, therefore, reinforced the findings of the previous reports [24,31,32].

Unexpectedly, the present study failed in demonstrating a significant fluctuation in the circulating level of EPC in our patients at three phases after acute IS. Interestingly, as compared with both Barthel Index and modified Ranking Scale score, NIHSS showed a notably better correlation with the circulating level of EPCs (E_1 _to E_3_) at 48 h after IS. Of importance is the fact that the circulating levels of EPCs were found to be inversely and significantly correlated to the severity of these three neurological scores at 48 h after acute IS. Our recent study [24] has revealed that a reduced circulating EPC level was significantly related to severe neurological impairment in patients after acute IS. Importantly, an increase in circulating levels of EPCs was found to be independently predictive of an improved 90-day MANE after IS in the current study. Interestingly, previous studies have shown that bone marrow-derived circulating EPCs play an essential role in repairing endothelial injury and participating directly in angiogenesis and vasculogenesis in systemic vascular beds [33,34]. Besides, contribution of EPCs in maintenance and repair of the cerebral vasculature has also been revealed in ischemia-related cerebrovascular dysfunction [35]. Therefore, our results strengthen the findings of previous studies [33-35]. Taken together, the results of the present study not only corroborate that of our recent report [24], they also encourage the possible use of the level circulating EPCs as a biomarker for risk stratification in patients after acute IS.

### Impact of EPO therapy effectively improves 90-day prognostic outcome after IS

Interestingly, while the neuroprotective mechanisms underlying EPO therapy have been well investigated in experimental studies [36] including the enhancement of mobilization of EPCs to circulation, attenuation of inflammatory response and cellular apoptosis, and reduction in oxidative stress [24,37-40], only a few clinical interventional studies [19,20,41] have been performed to further clarify the validity of this therapeutic option in improving the clinical outcome of patients after acute IS. On top of that, the data of these clinical observational studies [19,20,41] are too inconsistent to reach a significant conclusion. In the current study, we found an association between EPO therapy and reduction of the incidence of 90-day recurrent stroke. The most important finding in the present study is that EPO therapy was an independent predictor of improvement in 90-day MANE and in 90-day combined end point. Therefore, our findings, in addition to strengthening those of previous studies [19,41], highlight the therapeutic potential of EPO in patients after IS who are not suitable candidates for thrombolysis.

Contrary to the findings of our study, the results of one recent clinical trial did not find an additional benefit of EPO in improving clinical outcome of patients with acute IS undergoing tPA therapy as compared with placebo-controls [20]. However, subgroup analysis of the study showed that EPO therapy improved 90-day clinical outcome of the non-tPA patients. Thus, the findings in subgroup analysis of the study [20] support the results of our study. The reason accounting for the partially consistent results between this recent clinical study [20] and ours remain uncertain. However, there are some issues worthy of being addressed regarding that study [20]. First, as compared with the relative low dose of EPO adopted in our study, a very high EPO dosage used in that clinical trial [20] may raise other unidentified confounding effects such as a polycythemia and thrombosis event, thereby influencing patient outcomes. Second, of distinctive particularity was the majority of patients enrolled in that trial [20] were treated by tPA (a violation of their original protocol), which introduced another variable in assessing the benefit of EPO in improving patient outcome after IS, as tPA therapy may induce bleeding complications that outweigh the benefit of EPO treatment [13,14,20].

### Other independent predictors of 90-day MANE

The impact of blood pressure on clinical outcome after acute IS has been extensively investigated [42-44]. Excessive elevation, reduction, and variability in blood pressure have been reported to be independent prognostic predictors for poor clinical outcome after acute IS [43,44]. In the current study, another important finding is that SBP was an independent predictor of 90-day MANE. Further analysis demonstrated that SBP ≥135 mm Hg and ≤150 mm Hg were significantly associated with a favorable 90-day clinical outcome. Previous study [41] has also shown that satisfactory control of SBP within 140 to 150 mm Hg was the optimal therapy for improving clinical outcome after acute IS. Therefore, our findings corroborated those of previous studies [42-44].

Another notable finding in the current study is that total cholesterol level was also found to be an independent factor for predicting 90-day MANE in the current study. Conversely, HDL was strongly and independently associated with freedom from 90-day combined end point. Hypercholesterolemia and lower level of HDL-cholesterol have been identified as important contributing factors to the development of atherosclerosis and acute arterial obstructive syndrome [45,46]. Accordingly, our finding re-emphasizes the importance of serum cholesterol and HDL control in patients after acute IS.

### Study limitations

This study has limitations. First, practically, it is impossible to routinely perform intracranial angiographic examination for the acute IS patients. Therefore, the results of our study did not provide information to address the correlation between the circulating level of EPCs and angiogenesis, such as collateral formation in the brain infarct area. Second, because rather restrictive inclusion criteria were designed in the current study, some critical patients were excluded at the initial enrollment period. Thus, the 90-day mortality rate was relatively low in the current study. Therefore, the impact of EPO therapy on 90-day mortality could not be assessed. Finally, although a statistically significant relationship between EPO therapy and a reduction in the incidence of 90-day recurrent stroke was noted in the current investigation, this study did not give cutoff and define positive and negative predictive values as well as provide sensitivities and specificities of EPO on clinical outcome because this study was not actually designed to perform a prognostic study.

## Conclusions

EPO therapy significantly increased circulating EPC levels and was strongly associated with favorable 90-day clinical outcomes after IS. The results of this study, therefore, may encourage the application of EPO treatment in patients unsuitable for thrombolytic therapy after acute IS.

## Key messages

• EPO therapy in acute phase of IS was associated with an increase in circulating levels of EPCs at the convalescent phase of IS.

• EPO therapy was significantly associated with a reduction in the incidence of 90-day recurrent stroke.

• EPO therapy and increased circulating EPC (E_3_) levels were significantly and independently predictive of decreased 90-day MANE.

## Abbreviations

ACEI: angiotensin converting enzyme inhibitors; ADC: apparent diffusion coefficient; ARB: angiotensin II type I receptor blockers; DBP: diastolic blood pressure; ECCA: extra-cranial carotid artery; EPC: endothelial progenitor cell; EPO: erythropoietin; FITC: fluorescein isothiocyanate; HDL: high-density lipoprotein; IS: ischemic stroke; KDR: kinase insert domain-conjugating receptor; LDL: low-density lipoprotein; MANE: major adverse neurological event; MNC: mononuclear cell; MRI: Magnetic Resonance Imaging; NIHSS: National Institutes of Health Stroke Scale; PBS: phosphate buffered saline; PE: phycoerythrin; RBC: red blood cell; SBP: systolic blood pressure; tPA: tissue plasminogen activator; WBC: white blood cell.

## Competing interests

The authors declare that they have no competing interests.

## Authors' contributions

HKY and THT designed the experiment, drafted and performed laboratory work. CKS and SL were responsible for the laboratory assay and troubleshooting. HSL, SFC, CMY, TYT and MYL supervised clinical aspects and participated in patient recruitment. CWL and CHL participated in neurological function assessment. WNC and HKY participated in refinement of experiment protocol and coordination and helped in drafting the manuscript. All authors have read and approved the final manuscript.
